# Telemedicine in Patients Affected by Chronic Liver Disease: A Scoping Review of Clinical Outcomes and the Devices Evaluated

**DOI:** 10.3390/jcm12155128

**Published:** 2023-08-04

**Authors:** Paolo Capuano, Bethany Hileman, Stefano Tigano, Bianca Magro, Vincenzina Lo Re, Rosa Liotta, Marco Sciveres, Giusy Ranucci, Alessio Provenzani, Gaetano Burgio, Cesare Scardulla, Antonio Arcadipane, Gennaro Martucci

**Affiliations:** 1 Department of Anesthesia and Intensive Care, IRCCS-ISMETT (Istituto Mediterraneo per i Trapianti e Terapie ad Alta Specializzazione), 90133 Palermo, Italy; pcapuano@ismett.edu (P.C.); gburgio@ismett.edu (G.B.); aarcadipane@ismett.edu (A.A.); 2 University of Pittsburgh Medical Center (UPMC), 90133 Palermo, Italy; bmagro@ismett.edu (B.M.); vlore@ismett.edu (V.L.R.); rliotta@ismett.edu (R.L.); msciveres@ismett.edu (M.S.); granucci@ismett.edu (G.R.); aprovenzani@ismett.edu (A.P.); cscardulla@ismett.edu (C.S.); 3 School of Medicine, University of Pittsburgh, Pittsburgh, PA 15260, USA; hileman.bethany@medstudent.pitt.edu; 4 Department of Anesthesia and Intensive Care, A.O.U. Policlinico-San Marco, 95123 Catania, Italy; stefanotigano.st@gmail.com; 5 Hepatology Unit, Department for the Treatment and Study of Abdominal Diseases and Abdominal Transplantation, IRCCS-ISMETT (Istituto Mediterraneo per i Trapianti e Terapie ad Alta Specializzazione), 90133 Palermo, Italy; 6 Neurology Service, Department of Diagnostic and Therapeutic Services, IRCCS-ISMETT (Istituto Mediterraneo per i Trapianti e Terapie ad Alta Specializzazione), 90133 Palermo, Italy; 7 Pathology Service, Department of Diagnostic and Therapeutic Services, IRCCS-ISMETT (Istituto Mediterraneo per i Trapianti e Terapie ad Alta Specializzazione), 90133 Palermo, Italy; 8 Pediatric Center, IRCCS-ISMETT (Istituto Mediterraneo per i Trapianti e Terapie ad Alta Specializzazione), 90133 Palermo, Italy; 9 Pharmacy Service, IRCCS-ISMETT (Istituto Mediterraneo per i Trapianti e Terapie ad Alta Specializzazione), 90133 Palermo, Italy

**Keywords:** cirrhosis, telemonitoring, home-medicine, remote medicine, teleconsult, digital medicine, ascites, encephalopathy, outcomes

## Abstract

For patients with chronic liver disease (CLD), telemedicine is emerging as a useful tool to prevent liver decompensation or hospitalization, allowing access to and the decentralization of care, even for patients with limited resources. However, research and attendant evidence are still lacking; thus, this review aims to systematically explore the topic of telemonitoring for CLD to describe the currently used tools and clinical outcomes. The review was conducted by using key terms on PubMed/EMBASE and searching for observational studies or clinical trials (according to PRISMA recommendations) that were published between 6 April 2013 and 6 April 2023 to keep the technological framework limited to the last 10 years. The studies were described and grouped according to the aim of telemonitoring, the underlying disease, and the tools adopted to achieve remote monitoring. A total of 32 articles met the inclusion criteria. Of these, 11 articles report the successful use of a telehealth program to support and improve access to care in the management of HCV-related cirrhosis, eight articles examine the efficacy of telemedicine for remote monitoring interventions to prevent or decrease the risk of decompensation in high-risk patients, and five articles examine improvements in the physical performance and quality of life of cirrhotic patients through telehealth rehabilitation programs. Four studies were completed during the recent COVID-19 pandemic. Telehealth has the potential to provide and expand treatment access and reduce barriers to care for the most disadvantaged patients and might be able to reduce the need for hospital readmission for CLD, though most practice to test feasibility is still in the pilot stage.

## 1. Introduction

Patients with severe chronic liver disease (CLD) are at risk of recurrent complications, such as hepatic encephalopathy, ascites, hydro-electrolyte imbalance, spontaneous bacterial peritonitis, bleeding from esophageal varices, and hepatocellular carcinoma. These complications frequently occur during the transition from compensated to decompensated cirrhosis, a clinical worsening associated with a significant decrease in survival from 12 months to approximately 2 years [1]. Given the high number of patients affected by CLD, and the number of emerging causes, there is an urgent need to develop strategies to optimize the follow-up of such patients in order to reduce the risk of hospitalization in case of decompensation and to ensure the appropriate timing for liver transplantation [2,3].

The World Health Organization has defined telemedicine as the remote delivery of health care services by using modern tools, such as telecommunications and virtual technology, to provide care outside of hospital structures and facilities [4]. Telehealth was initially used to fill the gap in care for patients with inadequate access; in this light, it was widely implemented during the COVID-19 pandemic [5]. For patients with CLD pre-COVID pandemic, healthcare systems piloted telehealth programs to provide care to underserved populations, such as those living in remote areas or incarcerated persons, to ensure an early diagnosis for decompensation and allow for low-intensity treatments outside of the hospital environment [6].

However, despite the potential of telemedicine in CLD, evidence regarding efficacy is still lacking, and it is important to investigate its concrete effectiveness in patient care, also in view of the rapidly evolving technological scenario [7]. The currently available studies have a high level of heterogeneity in terms of the patient setting, the devices applied for remote monitoring, and the type of outcome considered. This scoping review aims to systematically explore the topic of telemonitoring in CLD to define the currently used tools and clinical outcomes that could be reproduced to hypothesize new and comprehensive prospective strategies.

## 2. Materials and Methods

This scoping review was conducted according to the recommendations of the Preferred Reporting Items for Systematic Reviews and Meta-Analyses (PRISMA) 2020 statement [8].

A systematic review of the PubMed and EMBASE databases was conducted by three independent reviewers (P.C., B.H., and S.T.). Publications from 6 April 2013 to 6 April 2023 were retrieved. The search strategy was as follows: (((cirrhosis) OR (“liver disease”)) OR (“liver transplant”)) AND (((((((“home monitoring”) OR (“remote monitoring”)) OR (telemonitoring)) OR (teleconsultation)) OR (televisit)) OR (telemedicine)) OR (telehealth)).

Two independent reviewers (P.C. and B.H.) screened the titles and abstracts of all identified studies. The inclusion criteria were (1) adult patients (over 18 years old); (2) being part of the chronic liver disease population (including cirrhosis, waiting list for liver transplantation, hepatitis C virus, non-alcoholic steatohepatitis (NASH), and non-alcoholic fatty liver disease (NAFLD) autoimmune hepatitis); (3) telemedicine, as the primary intervention of the study; and (4) an evaluation of a predefined clinical outcome. Studies were excluded if the telemedicine intervention was for physician education or if the study was descriptive in its design, incomplete, or did not report the relevant outcomes.

The included studies were evaluated by two independent reviewers (P.C. and S.T.) for the extraction of the following data: study characteristics (author, year of publication, design, aim, timeframe, and number of participants), patient condition, telemedicine intervention, and the relevant outcomes. Any conflicts were resolved by consensus after discussion with a third reviewer (G.M.).

Overall, the studies showed significant heterogeneity in the methods and outcomes adopted; consequently, we opted to provide a qualitative and descriptive summary of the current state of the art in the field rather than a meta-analysis.

For each included study, two independent reviewers (P.C. and B.H.) assessed the risk of bias using the RoB 2 tool for randomized trials [9]. The certainty of the evidence was assessed by four principal factors (risk of bias, inconsistency, indirectness, and imprecision) using the grading of recommendations assessment, development, and evaluations (GRADE) approach. The certainty of the evidence was rated from high (i.e., we are very confident that the true effect lies close to that of the effect estimate) to very low (i.e., we have very little confidence in the effect estimate; the true effect is likely to be substantially different). Any discrepancies in the bias assessment were recorded.

## 3. Results

**Figure 1:**
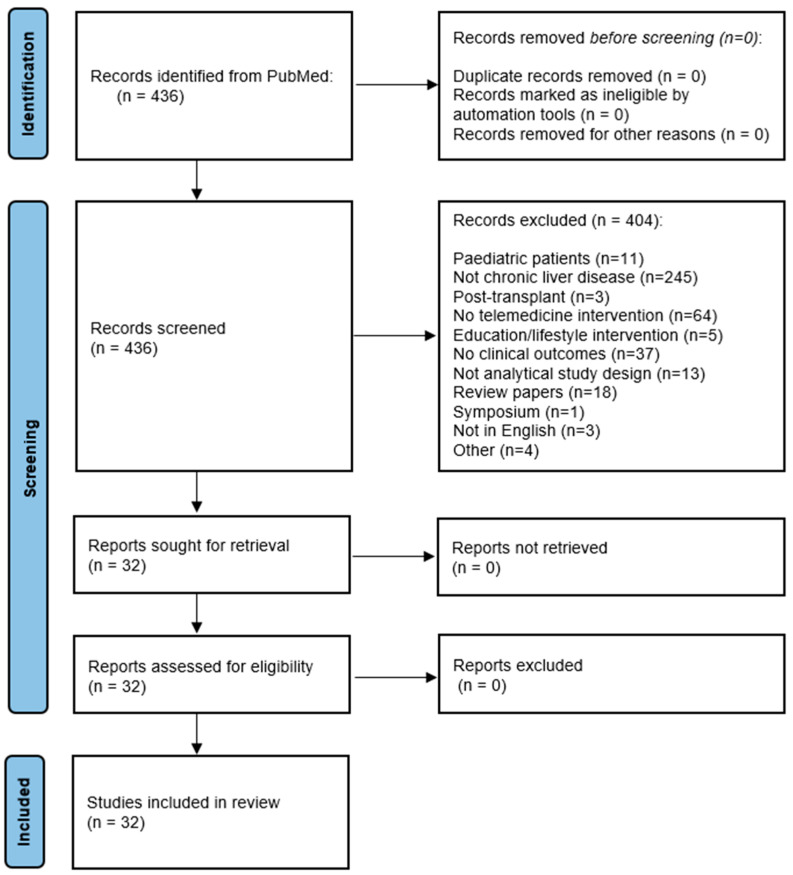
PRISMA flow diagram for study inclusion in the review.

A total of 436 articles were screened using the predefined terms. After critical revision using the inclusion criteria, 32 manuscripts met the criteria (Figure 1).

A total of 11 articles report on HCV treatment outcomes through telemedicine (Table 1); eight studies described the use of a telemedicine program for improving outcomes and preventing hospital readmission through remote teleconsultation or the use of a smartphone app and wireless technology to monitor and manage symptoms in patients at risk for decompensation.

Five studies describe the use of smartphone and smartwatch technology to improve patients’ physical function and quality of life, while three report on the benefit of an internet-based approach and of dietary intervention for lifestyle changes in patients with NAFLD.

Two studies describe the use of telehealth for screening in patients with HCC, and one study describes the use of telehealth for the management of patients with autoimmune hepatitis.

Finally, one study investigates the effects of telehealth on the liver transplant evaluation process, and one describes the cost-saving of an analytic-decision model developed for tracking patients with increasing ascites.

### 3.1. HCV Treatment Outcomes

Eleven articles report on HCV treatment outcomes through telehealth (Table 1), including remote specialist consultation, educational videos, and the support of primary care providers (five video telemedicine cases, four remote specialist consultations through teleconferences, one telephone consultation, and one paper-based referral followed by a fax or verbal discussion) [10,11,12,13,14,15,16,17,18,19,20].

All studies report a high rate of sustained virological response (SVR) to interferon-based or direct-acting antiviral therapies using a telehealth program to support and improve access to care in settings with limited access, such as rural or prison populations. No statistically significant difference in SVR rates was found between telehealth intervention and control groups in the historical general population.

### 3.2. Telemonitoring as a Tool for Improving Outcomes in Cirrhotic Patients at Risk of Decompensation

The use of remote tools for monitoring vital signs, together with educational programs for patients and their caregivers, can potentially improve symptom control and prevent the onset of decompensation in the cirrhotic patient (Table 2).

Thomson et al. [21] described an interactive voice call (IVR) system to manage and prevent hospitalization regarding cirrhotic patients. Patients received weekly IVR telephone calls for 3 months and responded to recorded messages via their touch-tone phones. They then received tailored self-management education on the basis of their response to recorded questions. In this pilot study, 62% of patients were hospitalized at least once during the follow-up period, and the results of the adjusted analysis showed that the independent predictors of hospitalization rates included self-reported weakness (based on questionnaire response) and a weekly weight increase of more than 5 lbs (2.3 kg).

Ganapathy et al. [22] explored the potential of a “Patient Buddy App” to prevent hospital readmissions secondary to hepatic encephalopathy, helping patients and caregivers to monitor symptoms, such as weight gain, adherence to medication, and daily sodium intake.

A smartphone app was also feasible to facilitate the management of ascites [23]. Through Bluetooth technology, the patient’s weight was monitored every weekday, and an alert was generated and sent to providers in the case of a weight change of ≥5 lbs in 1 week. During the study period, 17 readmissions occurred, but only 4 (24%) were related to ascites.

The readmission rate over 90 days was the clinical outcome explored by Khungar [24] to explore the effectiveness of remote monitoring for vital signs via wireless technology; a total of 15.8% of telehealth patients were readmitted within 30 days compared to 21% in the control population, with a contextually significant reduction in potentially preventable readmissions (PPRs) due to HE or volume overload (0% vs. 33.8%) at 90 days. Biometric parameters, such as blood pressure, peripheral oxygen saturation, and weight, were used to monitor symptoms of fluid overload, hepatic encephalopathy, bleeding, or infection. This study applied simple technology: 4G tablets, a wireless blood pressure monitor, a pulse oximeter, and a weighing scale. The patients kept tablets for 90 days, during which the parameters and symptom questionnaires were transmitted wirelessly to the reference center.

Su et al. [25] described the use of a virtual consultation program (SCAN-ECHO) in veterans with liver disease. A SCAN-ECHO visit, a tool for electronic consultation that combines real-time consultation and didactic learning, was associated with improved survival (a hazard ratio of 0.54 with a 95% confidence interval: 0.36–0.81, *p* = 0.003).

Recently, in 2023, Bajaj et al. [26] reported on the use of a smartphone app for the diagnosis of hepatic encephalopathy in cirrhotic patients and compared the results to healthy control patients. The EncephalApp, a streamlined version of the Stroop test, which evaluates cognitive flexibility and psychomotor speed, was used to detect covert hepatic encephalopathy (CHE). All patients with cirrhosis had worse EncephalApp test results than the controls, showing good face validity, test–retest reliability, and external validity regarding the app for the diagnosis of CHE. Similarly, in a recent trial, Luo et al. [27] described the use of EncephalApp and the electronic number connection test-A (eNCT-A) as potential home monitoring tools for cirrhotic patients at high risk of hepatic encephalopathy. The limits of these self-assessment tools are related to the interpretation of the results by the patient or caregivers and the patient’s performance improvement after repetition [28]. Preliminary experiences with wearable technologies based on portable electroencephalography (EEG) systems have been reported to monitor HE [29].

Oyelade et al. [30] evaluated the correlation between heart rate turbulence (HRT) parameters (defined as the variation in the length of the cardiac interbeat intervals after premature ventricular complexes) and mortality in cirrhotic patients. Twenty-four-hour electrocardiograph (ECG) recordings suitable for HRT analysis were obtained using a wireless Holter recorder, and the indices of HRT measured were turbulence onset (TO) and turbulence slope (TS). Patients were then followed up for 12 months. Turbulence onset was found to be strongly linked with mortality for Cox regression (Hazard ratio = 1.351, *p* < 0.05), independent of the MELD and Child-Pugh Score.

Similarly, Jansen et al. [31] reported the association between heart rate variability (HRV) and acute decompensation in cirrhotic patients. They performed wireless remote monitoring of HRV using the Isansys Lifetouch system (Isansys Limited, Oxford, UK). In order to better describe the variations in HRV in a time domain, the authors used (as a parameter) the standard deviation of all normal beat-beat intervals (SDNN). Then, through wireless remote HRV monitoring, SDNN was identified as an independent predictor of 90-day mortality.

**Table 2:** Studies on telemonitoring to prevent clinical decompensation in cirrhotic patients.

Study (Year) Design	Aim of the Study	Time Frame	Condition	Participants Exposed	Intervention/Exposure	Comparison	Outcomes	Sum of Results and Conclusion
Bloom, 2022 [7] Observational, prospective	To evaluate the cost and outcomes of current care compared to a telemonitoring system for ascites.	6 months	Cirrhosis	100	The telemonitoring system tracks patient weight remotely through Bluetooth-enabled scales and provides automated, early alerts to providers about weight changes.	100	Global costs. No. of hospital admissions. No. of office visits. No. of paracentesis.	A telemonitoring intervention is cost-saving for the management of cirrhotic ascites.
Ganapathy, 2017 [22] Observational, prospective	To evaluate the feasibility of using the Patient Buddy App and its impact on 30-day encephalopathy-related readmissions by engaging and educating cirrhotic inpatients and caregivers.	30 days post-discharge	Cirrhosis	40	The Patient Buddy focused on (a) medication and sodium intake adherence, (b) weight, and (c) orientation and cognition. The app automatically generates alerts for missed critical medications, missed measurements, significant changes to weight, or orientation/cognition scores, and contacts with on-call physicians or emergency services.	None	30-day readmission rate.	The use of Patient Buddy is feasible in recently discharged patients with cirrhosis and their caregivers.
Bajaj, 2015 [26] Observational, prospective	To validate the ability of the smartphone app EncephalApp, a streamlined version of the Stroop App, to detect covert hepatic encephalopathy.	NS	Cirrhosis and OHE	167	EncephalApp	114	EncephalApp and Paper&Pencil Test OffTime, OnTime, OffTime+OnTime, and number of runs required to complete five Off and On runs.	A smartphone app called EncephalApp has good face validity, test–retest reliability, and external validity for the diagnosis of CHE.
Oyelade, 2020 [30] Observational, prospective	To examine whether HRT parameters could predict mortality in cirrhotic patients.	12 months	Cirrhosis	40	24-h electrocardiograph (ECG) recordings suitable for HRT analysis were obtained using a wireless Holter recorder.	None	Mortality.	This study provides further evidence of autonomic dysfunction in cirrhosis, and suggests that HRT is a reliable alternative to HRV in patients with PVCs.
Jansen, 2019 [31] Observational, prospective	To validate wireless remote monitoring of HRV in AD patients, and establish whether HRV measurement is a surrogate for progression and inflammation, and if its measurement can determine prognosis in AD.	90-day follow-up between March 2013 and July 2015 in London and between February 2014 and January 2015 in Bonn	Cirrhosis	111	SDNN reflecting HRV using remote monitoring (Isansys Lifetouch) and/or Holter ECG recording.	NS	Disease progression and 90-day mortality.	SDNN predicted disease progression on repeat measures and appeared to be an independ- ent predictor of 90-day mortality (12 patients).
Thomson, 2015 [21] Observational, prospective	To investigate whether IVR monitoring can predict hospitalization and mortality in cirrhosis.	22 months	Cirrhosis	79	Patients using IVR hear recorded messages and respond to queries via their touch-tone phones. Based on their responses, they receive tailored self-management education. The focus of the questions was on factors previously identified as potential predictors of hospitalization or death among patients with cirrhosis.	None	Time to first hospital admission, hospitalization, and time to death.	IVR calls can be used to predict hospitalization in cirrhosis.
Luo, 2022 [27] Observational, retrospective	To compare the efficiency, convenience, accessibility, and acceptability of EncephalApp with that of eNCT-A for MHE screening in cirrhotic patients.	From January 2019 to January 2021	Cirrhosis	95	Both the EncephalApp Stroop test (EncephalApp) and eNCT-A were used for MHE screening.	150	The convenience, accessibility, and acceptability of PHES, EncephalApp and eNCT-A were respectively evaluated with the five-point Likert scale.	As with the EncephalApp, the eNCT-A will be a potential home monitoring and point-of-care tool for cirrhotic patients at high risk of MHE.
Khungar, 2017 [24] Observational, prospective (abstract)	To pilot a wireless mobile device monitoring system to detect early symptoms and signs, thereby preventing readmissions and keeping patients engaged and feeling cared for on a daily basis.	From 1 May 2010 to 7 July 2016	Cirrhosis	19	Biometric parameters, signs, and symptoms questionnaires were transmitted wirelessly. Physicians could intervene by phone or video chat.	143	N of readmissions.	A telehealth platform reduces readmissions for potentially preventable causes (HE and volume overload) and improves patient satisfaction.

OHE: overt hepatic encephalopathy; CHE: covert hepatic encephalopathy; HRT: heart rate turbulence; ECG: electrocardiograph; PVC: premature ventricular complexes; AD: acute decompensation; SDNN: standard deviation of all normal beat-beat intervals; IVR: interactive voice response; MHC: minimal hepatic encephalopathy; PHES: psychometric hepatic encephalopathy score; e-NCT-A: electronic number connection test-A; PPV: positive predictive value; NPV: negative predictive value.

### 3.3. Improving Physical Performance and Quality of Life

Rehabilitation programs can improve the quality of life of patients affected by chronic pathologies, including patients with liver disease. Home monitoring can help foster physical training and home-based physical activity programs (Table 3).

A 12-week home-based exercise training program for aerobic capacity was tested in cirrhotic patients [32]. The patients included in the study performed training exercises for 40 min per session four times a week. Smartphone technology (Garmin^®^ watch) was used to continuously monitor heart rate. Additionally, patients completed the translated and validated Thai version of the chronic liver disease questionnaire (CLDQ), a health-related quality-of-life questionnaire for liver disease patients. The authors found no significant differences regarding a 6-minute walk test, thigh muscle mass, or the hepatic venous pressure gradient, but the fatigue domain of the quality-of-life index was significantly improved in the home-based exercise training group when compared to the control group (*p* = 0.05, 95% CI 0.00 to 0.67).

Another home-based physical activity program (HB-PAP) over 12 weeks in patients with cirrhosis was based on a personal activity tracker (Charge HR, Fitbit; San Francisco, CA, USA) [33]. After a 2-week run-in period to document baseline physical activity (steps/day), the patients in the HB-PAP group (HB-PAP and dietary intervention) were compared to a control group with dietary intervention only. The authors observed significant differences in both daily step count and 6MWT, favoring the active group. The controls had a non-significant drop (418 ± 26 m vs. 327 ± 74 m), with a significant between-group difference.

Additionally, Duarte-Rojo et al. [34] tested a smartphone application (EL-FIT, Exercise and Liver FITness) on inpatients with end-stage liver disease. Through interaction with all app features (videos, perceived exertion, and gamification/motivational features), the patients improved their physical performance by 35%, and similar results were described by Stine et al. [35].

Finally, Motz et al. [36] reported the benefit of telehealth-based exercise training in patients with NASH during the COVID-19 pandemic.

### 3.4. Cost-Savings of Telemonitoring

Cirrhotic patients, as already mentioned, are at risk of frequent hospital readmissions, especially when decompensated. Telemonitoring can allow close follow-ups, reducing hospital admissions and the consequent healthcare costs [7]. An ascites evaluation using an analytic-decision model was developed, remotely tracking patients’ weight through Bluetooth-enabled scales and generating an automated early alert in case of significant weight changes. Despite the need for a specialized team to provide home-based assistance, the authors found that the cost of the standard of care for 100 patients with cirrhotic ascites over a 6-month period was USD 167,500 more expensive than telemonitoring (USD 1,221,500 vs. USD 1,054,000). The standard of care remained more expensive than telemonitoring, even when varying the parameter probabilities by ±10% and the outcome costs by ±20%.

**Table 3:** Studies reporting on the use of telehealth to improve self-training and quality of life.

Study (Year) Design	Aim of the Study	Time Frame	Condition	Participants Exposed	Intervention/Exposure	Comparison	Outcomes	Sum of Results and Conclusion
Duarte-Rojo, 2021 [34] Observational, prospective	To determine the feasibility of smartphone app and EL-FIT to facilitate exercise training in ESLD.	From July to December 2019	Cirrhosis	28	Daily steps, heart rate, and physical performance recorded by the smartphone application EL-FIT.	None	Physical performance	Showed that ESLD patients are able to use and interact with EL-FIT. This novel smartphone app has the potential of becoming an invaluable tool for home-based prehabilitation in LT candidates.
Chen, 2020 [33] RCT	To assess the benefits of an HB-PAP in patients with cirrhosis.	12 weeks	Cirrhosis	9	Personal activity tracker.	8	MELD-sodium 6MWT and CPET assessed changes in aerobic fitness. Different anthropometric measuring tools were used for skeletal muscle and adiposity assessment.	HB-PAP maintained physical performance and improved aerobic fitness according to 6MWT but not CPET, supporting the use of personal activity trackers to monitor/guide home-based prehabilitation programs in cirrhosis.
Sirisunhirun, 2022 [32] RCT	To evaluate the effect of a 12-week home-based exercise training program on aerobic capacity in cirrhotic patients	12 weeks	Cirrhosis	20	During home-based exercise training program, heart rate was continuously monitored using a Garmin^®^ watch.	20	6-minute walk test from baseline; difference in thigh muscle thickness, liver stiffness, spleen stiffness, and quality of life.	A 12-week moderate-intensity home-based exercise training program in compensated cirrhotic patients significantly improved the fatigue domain of the quality-of-life index without an increase in adverse events. However, no benefit in terms of aerobic capacity, thigh muscle mass, liver stiffness, and spleen stiffness was found.
Stine, 2023 [35] RCT	To determine whether a commercially available mHealth-delivered lifestyle intervention program can lead to clinically significant body weight loss in patients with NASH. We also determined the feasibility, acceptability, and safety of this widely accessible application.	16 weeks	NASH	20	NW, a mHealth lifestyle intervention program.	20	Change in body weight, feasibility, acceptability, and safety of Noom Weight.	NW is not only feasible, acceptable, and safe but also highly efficacious because this mHealth lifestyle intervention program led to significantly greater body weight loss than standard clinical care.
Motz, 2021 [36] Observational, prospective	To determine the feasibility of a directly supervised exercise training program delivered exclusively with telehealth to patients with NASH.	NS	NASH	3	A total of 20 weeks of moderate-intensity aerobic training 5 days a week under real-time direct supervision using an audio–visual telehealth platform. Aerobic training was completed by walking outdoors or using a home treadmill. Fitness activity trackers with heart rate monitors ensured exercise was completed at the prescribed intensity with real-time feedback from an exercise physiologist.	None	Completion rate of exercise sessions, adverse events, improvement in body weight and waist circumference, the mean relative reduction in liver fat measured by MRI-PDFF, mean reductions in hemoglobin A1c, aspartate aminotransferase, alanine aminotransferase, homeostatic model assessment for insulin resistance, the mean peak oxygen consumption (VO2 peak).	This proof-of-concept study found that supervised exercise training delivered via telehealth is feasible and safe in patients with NASH.

EL-FIT: Exercise and Liver FITness; ESLD: end-stage liver disease; LT: liver transplantation; HB-PAP: home-based physical activity program; 6MWT: six-minute walk test; CPET: cardiopulmonary exercise testing; NASH: non-alcoholic steatohepatitis; NW: Noom Weight; NS: not specified: MRI-PDFF: magnetic resonance imaging-proton density fat fraction.

## 4. Discussion

In this systematic review of telemedicine in CLD, we found a limited number of studies addressing this topic over the last 8 years, with these having three major focuses: (1) the efficacy of telemedicine and teleconferences in the management of HCV-related cirrhosis; (2) the potentially positive role of remote monitoring interventions in preventing or decreasing the risk of decompensation in high-risk patients; (3) devices and screening tools adopted in telemonitoring for cirrhosis that are simple applications for smartphones or smartwatches and easy-to-use vital sign monitors, including body weight.

Given the general urge of healthcare systems to move care from the hospital to peripheral care, this review is novel in recognizing the potential mid-term outcomes worth evaluating in larger prospective clinical trials. Today, thanks to the development of wireless and Bluetooth technology, and the improvement in audio and video connections, telemedicine allows access to and the decentralization of care, even for patients with limited resources. The limitation of the feasibility of a higher level of telemonitoring is that older age might reduce compliance with protocol follow-up. Additionally, on this issue, telemonitoring can overcome the contraindications since the “smart” tools are also tailored for an older population, but mainly, in the case of liver disease, the peculiarity of this population is that usually comprises young people during their active working and social life. For this reason, an impactful strategy to reduce hospitalization and improve follow-up might have an exponential positive impact on social and life experiences.

Telehealth has the potential to provide and expand treatment access and reduce the barriers to care for many disadvantaged patients, including those with a low socioeconomic status, disabilities, non-EU citizens, and older adults. Often these patients have limited access to treatment, also having to travel long distances to reach hospitals, losing days of work, and the added costs are often linked to the services of caregivers or child support [37]. Telemedicine can therefore contribute to a reduction in the patient’s travel burden, leading to a decentralization of care and improving the availability of resources for acute care. Though this aim has reduced relevance with the diffusion of the new DAA therapies, this model can be applied to the most severe patients at risk for rehospitalization and to patients on the liver transplantation waiting list to monitor their maintenance of an adequate level of fitness for transplantation [20].

More interestingly, telemedicine is emerging as a useful tool in chronic liver disease for monitoring symptoms such as ascites and encephalopathy. The monitoring of easy-to-perform tests, such as those monitoring increases in body weight, might contribute to preventing rehospitalizations, with a significant impact on financial costs.

In the above-cited study by Bloom et al. [7], the authors reported the cost savings of a telemonitoring program for cirrhotic ascites management, and it is, therefore, possible to hypothesize that the application of such a model to other conditions that are typical of the cirrhotic patient (hepatic encephalopathy, hydro-electrolyte imbalance, spontaneous bacterial peritonitis, and bleeding from esophageal varices) and associated complications, such as renal insufficiency or heart failure, may lead to improved outcomes.

However, beyond cost savings, there are other potential benefits when using a CLD telemonitoring program. The worsening of symptoms, hospital readmissions, and limited access to therapies are associated with poor quality of life [38]. Any intervention that could reduce the risk of decompensation would also likely increase the quality of life, in addition to rehabilitation and home-based physical activity programs. The benefit of physical exercise, through an increase in aerobic capacity and muscle mass, can also contribute to a decrease in hepatic vascular resistance, leading to a lowering in the hepatic venous pressure gradient (HVPG), and telehealth-based exercise and mobile health lifestyle programs offer potential therapeutic approaches for portal hypertension in patients with cirrhosis [39,40].

### The “Ideal” Telemedicine Approach

Telemedicine is one of the transitional care interventions that should be considered within a broad range of integrated services. It would certainly be very impactful for patients affected with severe cirrhosis and on a waiting list for an LT in order to prevent liver decompensation or hospitalization.

In recent years, sarcopenia and frailty have both played an important role in evaluating LTs and being predictors of liver-related complications and for this reason, we now have different scores to better stratify cirrhotic patients [41]. Most of the studies in this review reported an outpatient evaluation [41,42], and little is known in terms of a functional evaluation performed on at-home patients. This strongly suggests testing new technologies and digital advancements in the context of moving care from acute care hospitals to the patient’s home.

Home-integrated telemonitoring could include parameters through wireless systems to assess the functional reserve of cirrhotic patients and its modification or correlation with complication occurrence. All the monitoring systems that can be used to follow-up the patient frequently should be included in the medical organization. This could rely on easy-to-use platforms for the patients and caregivers and an available and well-structured platform for the physicians, nurses, and co-ordinators involved in the follow-up care. These technological aspects are limited in rural and remote areas, but technological improvement is currently so fast that we can hypothesize a rapid diffusion of web connections worldwide, and the use of medical telemonitoring may improve the economic conditions in rural areas and reduce the burden of the sick relative on the rest of the families that frequently have to afford long journeys to accompany the relative to the hub hospital. On the other hand, the development of easier-to-use tools at low production costs might spread such care in low-income countries and areas. However, to overcome these limitations regarding the availability of technical instrumentation, the organizational model should probably be adequately developed; in this light, artificial-intelligence-guided decisional algorithms may be the key to more personalized and population-centered medicine delivered at home and to peripheral areas.

This review has some limitations that reflect the tenuousness of home-based monitoring systems worldwide. First, when considering the variability and the limited number of studies, even for those with similar outcomes, it was not possible to carry out a meta-analysis. Second, the technological framework is rapidly moving forward, and the single pilot studies do not rely on solid platforms and organizational models; consequently, they usually tackle fragmentary topics and outcomes. Furthermore, since telemedicine is an emerging topic, it is possible that recent studies have been registered with MESH terms not included in our search and, therefore, were not found and included in the present review.

## 5. Conclusions

This systematic review highlights that telemedicine may be able to reduce the need for hospital readmission for CLD, though it has only been reported in terms of pilot activity to test its feasibility. By breaking down care barriers, telemedicine allows for closer adherence to therapies, the closer monitoring of symptoms, and improved patient self-engagement, thus improving their quality of life and reducing healthcare costs. Further studies carried out in adequate timeframes to reflect technological advancements should evaluate the use of telemedicine in organizational models.

## Figures and Tables

**Table 1:** Studies reporting on HCV treatment through telehealth.

Study (Year) Design	Aim of the Study	Time Frame	Condition	Participants Exposed	Intervention/Exposure	Comparison	Outcomes	Sum of Results and Conclusion
Case, 2019 [10] Observational, retrospective	To compare the rates of SVR between patients being treated in a CCVT telehepatology clinic versus a specialty care clinic (standard of care) in the era of DAAs.	Between 1 January 2014 and 31 December 2017	HCV infection	135	Telehepatology clinic consisted of clinic-to-clinic video telemedicine between the Denver VA Medical Center specialty clinic providers and patients located at one of six rural community-based outpatient clinics (CBOCs).	629	Sustained virological response (SVR) rates.	Hepatitis C treatment utilizing telehealth technologies to improve access to care does not negatively impact treatment outcomes compared with specialty care clinics in the era of DAAs.
Cooper, 2017 [11] Observational, retrospective	To evaluate whether telemedicine can facilitate the linkage of HCV-infected Canadians living in under-served and remote areas without access to HCV healthcare specialists.	Between January 2012 and August 2016	HCV infection	157	The patient and remote site TM nurse are linked by audio and video to The Ottawa Hospital site at which the HCV clinician, nurse, and allied health care providers are located.	1130	Pre-treatment access to biopsy, transient elastography (i.e., FibroScan), initiation and type of HCV antiviral treatment, and sustained virological response (SVR) rates.	TM patients initiated HCV therapy and achieved high SVR rates comparable to those obtained using traditional models of care.
Haridy, 2020 [12] Observational, retrospective	To evaluate outcomes of community-based treatment of hepatitis C virus (HCV) through a remote consultation process in the first 12 months.	Between 1 March 2016 and 28 February 2017	HCV infection	383	Remote consultation was defined as community-based treatment from a GP or hepatitis nurse in consultation with a tertiary center specialist through paper-based referral, fax, or verbal discussion, without in-person specialist consultation with the patient prior to treatment initiation.	None	Sustained virological response (SVR) rates.	Community-based management of HCV through remote specialist consultation can be an effective model of care.
Richter, 2022 [13] Observational, retrospective	To compare factors associated with HCV treatment success over the past decade in Israeli prisons, specifically the influence of DAAs and telemedicine.	From January 2010 to December 2020	HCV infection	139	The gastroenterology consultant and a nurse interview the prisoner using close-up cameras located at both sites with synchronous real-time patient management. The telemedicine facility consists of a computer with access to electronic patient data, including medical history, laboratory, and imaging results, as well as medical treatment.	134	Sustained virological response (SVR) rates.	Screening this high-risk population and using telemedicine for treatment can be an effective strategy for eliminating HCV from the prison population.
Rodrigues, 2021 [14] Observational, prospective	To assess qualitative and clinical outcomes in a clinical nurse consultant-led regional telehealth model.	From 1 April 2017 to 10 June 2020	HCV infection	24	Video-consult platform for clinical assessment of disease status, psychosocial assessment, clinic goal assessment.	None	Sustained virological response (SVR) rate at 12 weeks, cirrhosis monitoring and HCC surveillance, cost-effectiveness, overall patient satisfaction.	Clinical nurse consultant-led hepatitis C virus management via telehealth allows access to marginalized regional populations.
O’Brien, 2022 [15] Observational, retrospective	To provide further evidence on the effectiveness of telemedicine in HCV treatment in a large urban safety net hospital in the United States.	From 1 March 2019 to 23 July 2021	HCV infection	133	First, most appointments with HCV providers and all medication teaching visits with pharmacists were converted to telemedicine. Second, tests for liver fibrosis staging shifted from using in-person FibroScan^®^ tests to FibroSURE or FIB-4 laboratory tests that could be completed at the medical center or at another location based on patient preference. Third, medication was dispensed by mail delivery, if possible, to further reduce the patients’ need to come in person.	170	Appointment type, fibrosis staging method used, abdominal ultrasound for hepatocellular carcinoma screening, appointment attendance, treatment initiation, and SVR status. Only patients eligible for SVR tests were included in SVR status.	Appointments via telemedicine, transitioning to blood test-based fibrosis scoring, and medication delivery by mail can serve as tools to increase access to HCV care and successful HCV treatment completion even after COVID restrictions are lifted.
Papaluca, 2019 [16] Observational, prospective	To evaluate the feasibility and efficacy of a novel model of care for HCV patients.	13-month period	HCV infection	55 (+8)	The use of information technology, including telemedicine and a central electronic medical record, a centralized pharmacy distribution with real-time prisoner tracking, and federal government policy supporting prisoner access to DAAs.	13 (+8)	Sustained virological response (SVR) rates.	Hepatitis C treatment using a decentralized, nurse-led model of care is highly effective and can reach large numbers of prisoners.
Syed, 2020 [17] Observational, retrospective	To evaluate the efficacy and feasibility of HCV treatment in the Department of Corrections (DOC) through telemedicine.	From June 2015 to December 2019	HCV infection	870	The purpose of telemedicine visits was to document compliance, tolerance, side effects, duration, and response to treatment.	None	Sustained virological response (SVR) rates.	HCV treatment in the DOC through telemedicine is achievable and highly effective, with overall 97% SVR, irrespective of the underlying GT or DAA regimen used, and can eliminate HCV in this microenvironment and reduce the overall burden of HCV.
Wirth, 2022 [18] Observational, retrospective	First, to determine what percentage of patients during an ECHO session received DAA recommendations. Second, to analyze how U.S. Indian Country ECHO provides holistic care beyond the scope of HCV treatment. Third, to determine how ECHO served this subset of patients at increased risk of complications.	From February 2017 to March 2021	HCV infection	718	ECHO virtual telehealth clinics used Zoom Video Communications© (Zoom, San Jose, California) to connect PCPs serving AI/AN patients to a multi-disciplinary team of specialists, including physicians, pharmacists, and nurse practitioners, who provided comprehensive treatment recommendations.	None	Percentage of patients during an ECHO session who received DAA recommendations; number and type of recommendations that are beyond the scope of DAA prescription.	Most patients presenting at an Indian Country ECHO received recommendations for HCV treatment from their PCP, along with recommendations beyond the scope of HCV. Indian Country ECHO telehealth clinic provides comprehensive recommendations to effectively integrate evidence-based HCV treatment with holistic care at the primary care level.
Perez-Hernandez, 2021 [19] Observational, prospective	To implement a micro elimination program for HCV using DAAs with the support of a telemedicine program to minimize expenses.	Project started in January 2017	HCV infection	62	Virtual conferencing via telemedicine.	74	Cost estimations, sustained virological response (SVR) rates, treatment failure, adverse events.	With the aid of a telemedicine approach, significant savings were achieved by minimizing costs since nearly half of patients were distant from the study facility.
Arora, 2010 [20] Observational, prospective	To evaluate the efficacy and feasibility of the ECHO model.	Between 7 September 2004 and 29 February 2008 (genotype 1 or 4) or 15 August 2008 (genotype 2 or 3)	HCV infection	152	Community providers take part in weekly HCV clinics, called “knowledge networks,” by joining a video conference or calling into a teleconference line. Case-based discussions are supplemented with short didactic presentations by interdisciplinary experts to improve content knowledge.	84	Sustained virological response (SVR) rates.	The ECHO model is an effective way to treat HCV infection in underserved communities.

CCVT: clinic-to-clinic video telemedicine; DAA: direct-acting antivirals; CBOC: community-based outpatient clinics; SVR: sustained virological response; TM: telemedicine; GP: general practitioner; DOC: department of corrections; ECHO: Extension for Community Healthcare Outcomes; PCP: primary care providers; AI: American Indian; AN: Alaska Native.

## Data Availability

Data will be freely available after a motivated request.
